# 

**Published:** 1902-07-15

**Authors:** 


### ANNOUNCEMENTS.

### MICHIGAN STATE DENTAL ASSOCIATION.

The annual meeting of the Michigan State Dental Association was held at Grand Rapids, June 9-11, 1902, and the following officers were elected for the ensuing year: Pres. E. A. Honey; V-P., C. C. Noble; Secy., F. H. Essig; Treas., J. Ward House. We begin the proceedings in this issue.

### KENTUCKY STATE DENTAL ASSOCIATION.

The Kentucky State Dental Association held its annual meeting at Covington May 19-21, 1902, and elected the following officers: Pres. J. S. Cassidy; V-P., J. F. Clark; Sec'y, F. I. Gardner; Treas., F. R. Wilder. The next meeting will be held in Louisville, May, 1903.

